# 4-(4-Chloro­benzo­yl)-3-methyl-1-phenyl-1*H*-pyrazol-5-yl 4-chloro­benzoate

**DOI:** 10.1107/S1600536811048136

**Published:** 2011-11-23

**Authors:** Xiao-Xia Li, Zhong Chen

**Affiliations:** a Institute of Functional Materials, Jiangxi University of Finance & Economics, Nanchang 330013, People’s Republic of China

## Abstract

In the title compound, C_24_H_16_Cl_2_N_2_O_3_, the three benzene rings are twisted with respect to the central pyrazole ring, making dihedral angles of 71.56 (9) (4-chloro­benzo­yloxy), 57.55 (8) (4-chloro­benzo­yl) and 39.33 (1)° (phen­yl).

## Related literature

For the anti­bacterial and biological activity of 5-acyl­oxy­pyrazoles, see Bai *et al.* (2002 ▶); Varma (1999 ▶).
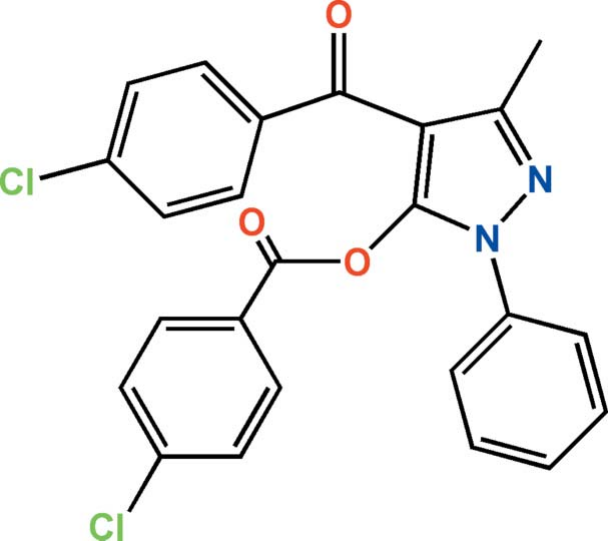


## Experimental

### 

#### Crystal data

C_24_H_16_Cl_2_N_2_O_3_
*M* *_r_* = 451.29
Monoclinic, 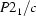

*a* = 11.4481 (4) Å
*b* = 29.4169 (13) Å
*c* = 6.4120 (3) Å
β = 99.669 (3)°
*V* = 2128.68 (16) Å^3^
*Z* = 4
Mo *K*α radiation
μ = 0.33 mm^−1^
*T* = 296 K
0.23 × 0.18 × 0.16 mm

#### Data collection

Bruker APEXII CCD diffractometer
Absorption correction: multi-scan (*SADABS*; Bruker, 2007 ▶) *T*_min_ = 0.930, *T*_max_ = 0.948
16531 measured reflections
3765 independent reflections
1884 reflections with *I* > 2σ(*I*)
*R*_int_ = 0.067

#### Refinement

*R*[*F* ^2^ > 2σ(*F* ^2^)] = 0.045
*wR*(*F* ^2^) = 0.093
*S* = 1.03
3765 reflections
281 parameters
H-atom parameters constrained
Δρ_max_ = 0.15 e Å^−3^
Δρ_min_ = −0.19 e Å^−3^

### 

Data collection: *APEX2* (Bruker, 2007 ▶); cell refinement: *SAINT* (Bruker, 2007 ▶); data reduction: *SAINT*; program(s) used to solve structure: *SHELXS97* (Sheldrick, 2008 ▶); program(s) used to refine structure: *SHELXL97* (Sheldrick, 2008 ▶); molecular graphics: *SHELXTL* (Sheldrick, 2008 ▶); software used to prepare material for publication: *SHELXTL*.

## Supplementary Material

Crystal structure: contains datablock(s) I, global. DOI: 10.1107/S1600536811048136/vm2134sup1.cif

Structure factors: contains datablock(s) I. DOI: 10.1107/S1600536811048136/vm2134Isup2.hkl

Supplementary material file. DOI: 10.1107/S1600536811048136/vm2134Isup3.cml

Additional supplementary materials:  crystallographic information; 3D view; checkCIF report

## Figures and Tables

**Fig. 1.**
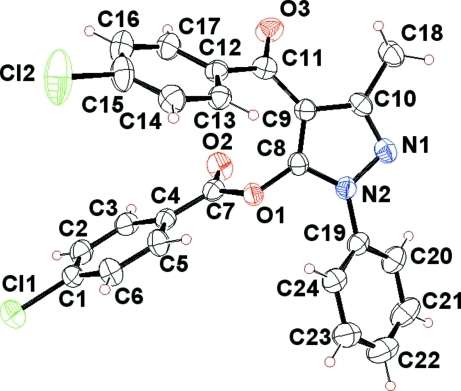
The title molecule with the displacement ellipsoids shown at the 30% probability level.

## supplementary crystallographic information

### Comment

5-Acyloxypyrazoles have received considerable attention due to their high antibacterial and biological activities (Bai *et al.*, 2002, Varma, 1999). As part of our ongoing search for  biologically active molecules, the title compound was synthesized and characterized by X-ray diffraction (Fig. 1).

The three phenyl rings (C1—C6, r.m.s. deviation = 0.0047 Å; C12—C17, r.m.s. deviation = 0.0061 Å; C19—C24, r.m.s. deviation = 0.0045 Å) are twisted with respect to the central pyrazole ring (N1,2/C8—C10, r.m.s. deviation = 0.0065 Å) making dihedral angles of 71.56 (9)°, 57.55 (8)° and 39.33 (1)°, respectively. As expected, there are no classic hydrogen bonds in the structure.

### Experimental

1.74 g of 1-phenyl-3-methyl-5-pyrazolone was dissolved in 10 ml dioxane by heating to 80 °C, and 1.5 g of calcium hydroxide was added, then 2 ml of 4-chlorobenzoylchloride was added dropwise within 20 min. After refluxing for 2 h, the reaction mixture was poured into 30 ml diluted hydrochloric acid (3 N). A cream colored powder was obtained by filtration, and used for recrystallization from ethanol to give colorless needle crystals.

### Refinement

All H atoms were placed in calculated positions, with the carrier atom-H distances = 0.96 Å for the methyl and 0.93 Å for aryl, and refined as riding, with the *U*_iso_(H) = 1.5Ueq(C) for methyl groups and 1.2Ueq(C) for aryl groups.

### Crystal data

**Table:**

C_24_H_16_Cl_2_N_2_O_3_	*F*(000) = 928
*M**_r_* = 451.29	*D*_x_ = 1.408 Mg m^−^^3^
Monoclinic, *P*2_1_/*c*	Mo *K*α radiation, λ = 0.71073 Å
Hall symbol: -P 2ybc	Cell parameters from 1569 reflections
*a* = 11.4481 (4) Å	θ = 2.8–19.2°
*b* = 29.4169 (13) Å	µ = 0.33 mm^−^^1^
*c* = 6.4120 (3) Å	*T* = 296 K
β = 99.669 (3)°	Needle, colorless
*V* = 2128.68 (16) Å^3^	0.23 × 0.18 × 0.16 mm
*Z* = 4	

### Data collection

**Table:**

Bruker APEXII CCD diffractometer	3765 independent reflections
Radiation source: fine-focus sealed tube	1884 reflections with *I* > 2σ(*I*)
graphite	*R*_int_ = 0.067
φ and ω scans	θ_max_ = 25.0°, θ_min_ = 1.4°
Absorption correction: multi-scan (*SADABS*; Bruker, 2007)	*h* = −13→13
*T*_min_ = 0.930, *T*_max_ = 0.948	*k* = −35→34
16531 measured reflections	*l* = −7→7

### Refinement

**Table:**

Refinement on *F*^2^	Primary atom site location: structure-invariant direct methods
Least-squares matrix: full	Secondary atom site location: difference Fourier map
*R*[*F*^2^ > 2σ(*F*^2^)] = 0.045	Hydrogen site location: inferred from neighbouring sites
*wR*(*F*^2^) = 0.093	H-atom parameters constrained
*S* = 1.03	*w* = 1/[σ^2^(*F*_o_^2^) + (0.0199*P*)^2^ + 0.5*P*] where *P* = (*F*_o_^2^ + 2*F*_c_^2^)/3
3765 reflections	(Δ/σ)_max_ < 0.001
281 parameters	Δρ_max_ = 0.15 e Å^−^^3^
0 restraints	Δρ_min_ = −0.19 e Å^−^^3^

### Special details

**Table:**

Geometry. All e.s.d.'s (except the e.s.d. in the dihedral angle between two l.s. planes) are estimated using the full covariance matrix. The cell e.s.d.'s are taken into account individually in the estimation of e.s.d.'s in distances, angles and torsion angles; correlations between e.s.d.'s in cell parameters are only used when they are defined by crystal symmetry. An approximate (isotropic) treatment of cell e.s.d.'s is used for estimating e.s.d.'s involving l.s. planes.
Refinement. Refinement of *F*^2^ against ALL reflections. The weighted *R*-factor *wR* and goodness of fit *S* are based on *F*^2^, conventional *R*-factors *R* are based on *F*, with *F* set to zero for negative *F*^2^. The threshold expression of *F*^2^ > σ(*F*^2^) is used only for calculating *R*-factors(gt) *etc*. and is not relevant to the choice of reflections for refinement. *R*-factors based on *F*^2^ are statistically about twice as large as those based on *F*, and *R*- factors based on ALL data will be even larger.

### Fractional atomic coordinates and isotropic or equivalent isotropic displacement parameters (Å^2^)

**Table:**

	*x*	*y*	*z*	*U*_iso_*/*U*_eq_	
Cl1	0.69348 (7)	0.00005 (3)	0.62066 (14)	0.0918 (3)	
C1	0.7963 (2)	−0.02529 (11)	0.8140 (5)	0.0605 (8)	
C2	0.8162 (3)	−0.00773 (10)	1.0144 (6)	0.0689 (9)	
H2A	0.7739	0.0175	1.0469	0.083*	
C3	0.8991 (2)	−0.02759 (10)	1.1679 (5)	0.0609 (8)	
H3A	0.9118	−0.0160	1.3048	0.073*	
C4	0.9635 (2)	−0.06468 (9)	1.1194 (5)	0.0499 (7)	
C5	0.9413 (2)	−0.08216 (10)	0.9159 (5)	0.0605 (8)	
H5A	0.9832	−0.1074	0.8820	0.073*	
C6	0.8580 (3)	−0.06247 (10)	0.7642 (5)	0.0672 (9)	
H6A	0.8435	−0.0743	0.6279	0.081*	
C7	1.0529 (2)	−0.08436 (9)	1.2859 (5)	0.0530 (8)	
C8	1.1952 (2)	−0.14311 (9)	1.3390 (4)	0.0506 (7)	
C9	1.1780 (2)	−0.17194 (9)	1.4968 (4)	0.0501 (7)	
C10	1.2931 (3)	−0.18817 (9)	1.5767 (4)	0.0550 (8)	
C11	1.0674 (3)	−0.18198 (9)	1.5742 (5)	0.0587 (8)	
C12	0.9553 (3)	−0.18520 (9)	1.4215 (5)	0.0533 (7)	
C13	0.9556 (3)	−0.19948 (9)	1.2166 (5)	0.0580 (8)	
H13A	1.0271	−0.2058	1.1722	0.070*	
C14	0.8509 (3)	−0.20449 (10)	1.0771 (5)	0.0695 (9)	
H14A	0.8512	−0.2146	0.9398	0.083*	
C15	0.7463 (3)	−0.19438 (12)	1.1436 (6)	0.0824 (11)	
C16	0.7438 (3)	−0.18042 (11)	1.3472 (7)	0.0853 (11)	
H16A	0.6721	−0.1739	1.3908	0.102*	
C17	0.8479 (3)	−0.17622 (10)	1.4846 (5)	0.0679 (9)	
H17A	0.8467	−0.1671	1.6231	0.081*	
C18	1.3268 (3)	−0.22254 (10)	1.7484 (5)	0.0731 (9)	
H18A	1.4014	−0.2360	1.7344	0.110*	
H18C	1.2671	−0.2457	1.7372	0.110*	
H18B	1.3334	−0.2079	1.8837	0.110*	
C19	1.3721 (3)	−0.11677 (9)	1.1883 (5)	0.0533 (7)	
C20	1.4818 (3)	−0.09849 (11)	1.2704 (5)	0.0726 (9)	
H20A	1.5152	−0.1028	1.4115	0.087*	
C21	1.5397 (3)	−0.07371 (13)	1.1369 (7)	0.0947 (12)	
H21A	1.6136	−0.0613	1.1884	0.114*	
C22	1.4902 (4)	−0.06699 (12)	0.9297 (7)	0.0928 (12)	
H22A	1.5301	−0.0498	0.8422	0.111*	
C23	1.3817 (3)	−0.08555 (11)	0.8510 (6)	0.0753 (9)	
H23A	1.3482	−0.0810	0.7101	0.090*	
C24	1.3226 (3)	−0.11086 (10)	0.9802 (5)	0.0602 (8)	
H24A	1.2496	−0.1239	0.9270	0.072*	
O1	1.11813 (15)	−0.11725 (6)	1.2022 (3)	0.0523 (5)	
O2	1.07060 (18)	−0.07494 (7)	1.4689 (3)	0.0757 (6)	
O3	1.06779 (17)	−0.18818 (7)	1.7646 (3)	0.0763 (6)	
N1	1.3736 (2)	−0.17134 (8)	1.4732 (4)	0.0589 (7)	
N2	1.3115 (2)	−0.14249 (8)	1.3265 (4)	0.0524 (6)	
Cl2	0.61559 (9)	−0.19901 (5)	0.9673 (2)	0.1547 (6)	

### Atomic displacement parameters (Å^2^)

**Table:**

	*U*^11^	*U*^22^	*U*^33^	*U*^12^	*U*^13^	*U*^23^
Cl1	0.0762 (6)	0.0896 (6)	0.1072 (7)	0.0202 (5)	0.0090 (5)	0.0366 (6)
C1	0.0492 (18)	0.055 (2)	0.077 (2)	0.0040 (15)	0.0114 (18)	0.0182 (19)
C2	0.064 (2)	0.053 (2)	0.094 (3)	0.0148 (17)	0.026 (2)	0.007 (2)
C3	0.067 (2)	0.053 (2)	0.067 (2)	0.0022 (16)	0.0214 (18)	−0.0036 (17)
C4	0.0458 (17)	0.0458 (18)	0.060 (2)	0.0009 (14)	0.0139 (15)	−0.0008 (16)
C5	0.0576 (19)	0.057 (2)	0.066 (2)	0.0128 (15)	0.0060 (17)	−0.0045 (17)
C6	0.066 (2)	0.066 (2)	0.066 (2)	0.0115 (18)	0.0011 (18)	0.0014 (18)
C7	0.0514 (19)	0.0457 (19)	0.064 (2)	−0.0031 (15)	0.0158 (18)	−0.0042 (18)
C8	0.0475 (19)	0.0554 (19)	0.0473 (19)	0.0039 (15)	0.0028 (15)	−0.0034 (16)
C9	0.0492 (18)	0.0528 (18)	0.0465 (19)	−0.0039 (15)	0.0025 (15)	−0.0007 (15)
C10	0.064 (2)	0.0504 (19)	0.0459 (19)	0.0000 (16)	−0.0031 (16)	−0.0006 (15)
C11	0.069 (2)	0.055 (2)	0.052 (2)	−0.0016 (16)	0.0106 (18)	−0.0029 (17)
C12	0.0540 (19)	0.0517 (19)	0.054 (2)	−0.0058 (15)	0.0089 (16)	0.0002 (16)
C13	0.0527 (18)	0.062 (2)	0.058 (2)	−0.0050 (15)	0.0068 (17)	−0.0002 (17)
C14	0.068 (2)	0.071 (2)	0.066 (2)	−0.0045 (18)	0.0012 (19)	−0.0051 (17)
C15	0.055 (2)	0.085 (3)	0.098 (3)	0.0035 (19)	−0.016 (2)	−0.016 (2)
C16	0.056 (2)	0.088 (3)	0.111 (3)	0.0097 (19)	0.011 (2)	−0.014 (2)
C17	0.062 (2)	0.069 (2)	0.074 (2)	0.0028 (17)	0.015 (2)	−0.0066 (18)
C18	0.073 (2)	0.070 (2)	0.070 (2)	0.0026 (17)	−0.0050 (18)	0.0079 (18)
C19	0.0528 (19)	0.0463 (18)	0.064 (2)	−0.0010 (15)	0.0180 (17)	−0.0075 (16)
C20	0.056 (2)	0.081 (2)	0.080 (2)	−0.0097 (18)	0.0120 (19)	−0.011 (2)
C21	0.069 (2)	0.104 (3)	0.116 (4)	−0.034 (2)	0.031 (3)	−0.017 (3)
C22	0.102 (3)	0.081 (3)	0.107 (3)	−0.020 (2)	0.050 (3)	−0.001 (2)
C23	0.091 (3)	0.064 (2)	0.077 (2)	−0.001 (2)	0.031 (2)	0.0006 (19)
C24	0.0638 (19)	0.057 (2)	0.062 (2)	−0.0030 (16)	0.0170 (18)	−0.0064 (17)
O1	0.0490 (11)	0.0569 (12)	0.0507 (12)	0.0087 (10)	0.0076 (10)	0.0031 (10)
O2	0.0890 (15)	0.0802 (16)	0.0559 (14)	0.0158 (12)	0.0069 (13)	−0.0154 (12)
O3	0.0765 (14)	0.0987 (17)	0.0542 (14)	−0.0110 (12)	0.0128 (12)	0.0034 (13)
N1	0.0559 (15)	0.0540 (16)	0.0626 (17)	0.0024 (13)	−0.0020 (14)	0.0010 (13)
N2	0.0463 (15)	0.0524 (15)	0.0580 (16)	0.0006 (12)	0.0073 (13)	−0.0002 (13)
Cl2	0.0745 (7)	0.1987 (13)	0.1694 (12)	0.0168 (7)	−0.0416 (7)	−0.0614 (10)

### Geometric parameters (Å, °)

**Table:**

Cl1—C1	1.728 (3)	C13—C14	1.377 (4)
C1—C2	1.368 (4)	C13—H13A	0.9300
C1—C6	1.369 (4)	C14—C15	1.369 (4)
C2—C3	1.377 (4)	C14—H14A	0.9300
C2—H2A	0.9300	C15—C16	1.374 (4)
C3—C4	1.381 (3)	C15—Cl2	1.723 (3)
C3—H3A	0.9300	C16—C17	1.363 (4)
C4—C5	1.386 (4)	C16—H16A	0.9300
C4—C7	1.468 (4)	C17—H17A	0.9300
C5—C6	1.370 (3)	C18—H18A	0.9600
C5—H5A	0.9300	C18—H18C	0.9600
C6—H6A	0.9300	C18—H18B	0.9600
C7—O2	1.189 (3)	C19—C24	1.370 (4)
C7—O1	1.384 (3)	C19—C20	1.386 (4)
C8—N2	1.348 (3)	C19—N2	1.430 (3)
C8—C9	1.359 (3)	C20—C21	1.376 (4)
C8—O1	1.366 (3)	C20—H20A	0.9300
C9—C10	1.414 (3)	C21—C22	1.368 (4)
C9—C11	1.466 (4)	C21—H21A	0.9300
C10—N1	1.319 (3)	C22—C23	1.373 (4)
C10—C18	1.496 (4)	C22—H22A	0.9300
C11—O3	1.233 (3)	C23—C24	1.374 (4)
C11—C12	1.481 (4)	C23—H23A	0.9300
C12—C13	1.380 (4)	C24—H24A	0.9300
C12—C17	1.383 (4)	N1—N2	1.373 (3)
			
C2—C1—C6	120.6 (3)	C15—C14—H14A	120.5
C2—C1—Cl1	119.8 (3)	C13—C14—H14A	120.5
C6—C1—Cl1	119.6 (3)	C14—C15—C16	121.3 (3)
C1—C2—C3	119.8 (3)	C14—C15—Cl2	119.2 (3)
C1—C2—H2A	120.1	C16—C15—Cl2	119.5 (3)
C3—C2—H2A	120.1	C17—C16—C15	119.0 (3)
C2—C3—C4	120.2 (3)	C17—C16—H16A	120.5
C2—C3—H3A	119.9	C15—C16—H16A	120.5
C4—C3—H3A	119.9	C16—C17—C12	121.2 (3)
C3—C4—C5	119.0 (3)	C16—C17—H17A	119.4
C3—C4—C7	118.7 (3)	C12—C17—H17A	119.4
C5—C4—C7	122.2 (3)	C10—C18—H18A	109.5
C6—C5—C4	120.5 (3)	C10—C18—H18C	109.5
C6—C5—H5A	119.8	H18A—C18—H18C	109.5
C4—C5—H5A	119.8	C10—C18—H18B	109.5
C5—C6—C1	119.8 (3)	H18A—C18—H18B	109.5
C5—C6—H6A	120.1	H18C—C18—H18B	109.5
C1—C6—H6A	120.1	C24—C19—C20	121.4 (3)
O2—C7—O1	122.1 (3)	C24—C19—N2	120.4 (3)
O2—C7—C4	127.4 (3)	C20—C19—N2	118.2 (3)
O1—C7—C4	110.4 (3)	C21—C20—C19	118.0 (3)
N2—C8—C9	108.7 (2)	C21—C20—H20A	121.0
N2—C8—O1	119.5 (3)	C19—C20—H20A	121.0
C9—C8—O1	131.8 (3)	C22—C21—C20	121.1 (3)
C8—C9—C10	103.7 (3)	C22—C21—H21A	119.5
C8—C9—C11	128.2 (3)	C20—C21—H21A	119.5
C10—C9—C11	127.9 (3)	C21—C22—C23	120.1 (4)
N1—C10—C9	112.2 (3)	C21—C22—H22A	120.0
N1—C10—C18	120.5 (3)	C23—C22—H22A	120.0
C9—C10—C18	127.1 (3)	C24—C23—C22	120.0 (3)
O3—C11—C9	120.3 (3)	C24—C23—H23A	120.0
O3—C11—C12	120.3 (3)	C22—C23—H23A	120.0
C9—C11—C12	119.4 (3)	C19—C24—C23	119.4 (3)
C13—C12—C17	118.8 (3)	C19—C24—H24A	120.3
C13—C12—C11	120.6 (3)	C23—C24—H24A	120.3
C17—C12—C11	120.6 (3)	C8—O1—C7	118.2 (2)
C14—C13—C12	120.6 (3)	C10—N1—N2	104.6 (2)
C14—C13—H13A	119.7	C8—N2—N1	110.7 (2)
C12—C13—H13A	119.7	C8—N2—C19	129.1 (2)
C15—C14—C13	119.1 (3)	N1—N2—C19	120.2 (2)
			
C6—C1—C2—C3	0.0 (4)	C12—C13—C14—C15	1.1 (4)
Cl1—C1—C2—C3	−179.2 (2)	C13—C14—C15—C16	−1.6 (5)
C1—C2—C3—C4	1.0 (4)	C13—C14—C15—Cl2	178.1 (2)
C2—C3—C4—C5	−1.5 (4)	C14—C15—C16—C17	0.6 (5)
C2—C3—C4—C7	178.7 (2)	Cl2—C15—C16—C17	−179.0 (2)
C3—C4—C5—C6	1.0 (4)	C15—C16—C17—C12	0.8 (5)
C7—C4—C5—C6	−179.2 (3)	C13—C12—C17—C16	−1.3 (4)
C4—C5—C6—C1	0.0 (4)	C11—C12—C17—C16	−177.9 (3)
C2—C1—C6—C5	−0.5 (4)	C24—C19—C20—C21	0.6 (4)
Cl1—C1—C6—C5	178.7 (2)	N2—C19—C20—C21	−179.9 (3)
C3—C4—C7—O2	7.8 (4)	C19—C20—C21—C22	0.5 (5)
C5—C4—C7—O2	−171.9 (3)	C20—C21—C22—C23	−0.8 (6)
C3—C4—C7—O1	−172.5 (2)	C21—C22—C23—C24	0.1 (5)
C5—C4—C7—O1	7.8 (3)	C20—C19—C24—C23	−1.3 (4)
N2—C8—C9—C10	0.1 (3)	N2—C19—C24—C23	179.2 (2)
O1—C8—C9—C10	179.6 (3)	C22—C23—C24—C19	1.0 (4)
N2—C8—C9—C11	177.1 (3)	N2—C8—O1—C7	−120.0 (3)
O1—C8—C9—C11	−3.5 (5)	C9—C8—O1—C7	60.6 (4)
C8—C9—C10—N1	−1.2 (3)	O2—C7—O1—C8	7.8 (4)
C11—C9—C10—N1	−178.2 (3)	C4—C7—O1—C8	−171.9 (2)
C8—C9—C10—C18	−177.5 (3)	C9—C10—N1—N2	1.7 (3)
C11—C9—C10—C18	5.5 (5)	C18—C10—N1—N2	178.3 (2)
C8—C9—C11—O3	−142.7 (3)	C9—C8—N2—N1	0.9 (3)
C10—C9—C11—O3	33.6 (4)	O1—C8—N2—N1	−178.6 (2)
C8—C9—C11—C12	38.1 (4)	C9—C8—N2—C19	−178.7 (2)
C10—C9—C11—C12	−145.7 (3)	O1—C8—N2—C19	1.8 (4)
O3—C11—C12—C13	−149.5 (3)	C10—N1—N2—C8	−1.6 (3)
C9—C11—C12—C13	29.7 (4)	C10—N1—N2—C19	178.0 (2)
O3—C11—C12—C17	27.1 (4)	C24—C19—N2—C8	−40.2 (4)
C9—C11—C12—C17	−153.6 (3)	C20—C19—N2—C8	140.2 (3)
C17—C12—C13—C14	0.3 (4)	C24—C19—N2—N1	140.2 (3)
C11—C12—C13—C14	177.0 (3)	C20—C19—N2—N1	−39.3 (3)
